# Studies on *Acanthocheilonema viteae *cystatin: Genomic organization, promoter studies and expression in *Caenorhabditis elegans*

**DOI:** 10.1186/1475-2883-4-9

**Published:** 2005-08-09

**Authors:** Smitha Pillai, Bernd H Kalinna, Eva Liebau, Susanne Hartmann, Franz Theuring, Richard Lucius

**Affiliations:** 1 Department of Molecular Parasitology, Institute of Biology, Humboldt University Berlin, 10115 Berlin, Germany; 2 Department of Biochemistry, Bernhard Nocht Institute for Tropical Medicine, 20359 Hamburg, Germany; 3 Institute for Pharmacology and Toxicology, Charitée, 10115 Berlin, Germany

**Keywords:** *Caenorhabditis elegans*, *Acanthocheilonema viteae*, *cystatin*, *promoter*, *expression*, *localization*

## Abstract

Cystatins are reversible, tightly binding inhibitors of cysteine proteases. Filarial cystatins have been ascribed immunomodulatory properties and have been implicated in protective immunity. To continue exploration of this potential, here we have determined the sequence, structure and genomic organization of the cystatin gene locus of *A. viteae*. The gene is composed of 4 exons separated by 3 introns and spans ~2 kb of genomic DNA. The upstream genomic sequence contains transcriptional factor binding sites such as AP-1 and NF-Y, an inverted CCAAT sequence, and a TATA box. To investigate sites of cystatin expression, *Caenorhabditis elegans *worms were transformed by microinjection with the putative promoter region and the first exon of the *A. viteae *cystatin gene fused to the reporter GFP. In transgenic worms fluorescence was observed in the pharyngeal and rectal gland cells suggesting that cystatin is secreted. Additionally, *A. viteae *cystatin was expressed in *C. elegans *to explore its potential as an expression system for filarial genes.

## 1. Introduction

Filarial nematodes reside among others in the lymphatic vessels or the subcutis of their vertebrate hosts, where they often persist for many years in spite of an array of immune effector mechanisms. The persistence of the parasites has in part been attributed to the fact that they interfere with the regulation of immune responses and induce anti-inflammatory immune reactions [1]. One of the parasite-derived molecules described in this context is the filarial excretory/secretory protein cystatin. Cystatins are reversible, tightly binding inhibitors of cysteine proteases [2]. Cystatin of *Onchocerca volvulus *was first described by Lustigmann et al. [3]. Since then studies on cystatin of other parasitic nematodes such as the rodent filaria *Acanthocheilonema viteae *[4], *Brugia malayi *[5,6], *Litomosoides sigmodontis *[7], *Nippostrongylus brasiliensis *[8] and *O. volvulus *[9] have revealed that it is a modulator of the host immune response. Cystatins of parasitic nematodes have been shown to interfere with the immune response by inhibition of proteases and induction of cytokines [10-13] and can thus be considered as a major pathogenicity factor. Cystatin of *O. volvulus *[14] and *A. viteae *(unpublished) have also been tested as vaccine antigens.

Functional studies on cystatin so far have relied on recombinant proteins expressed in *E. coli *[4]. The investigation of its role could however be simplified by over-expression, knock-down of the protein by RNAi [15] or production of knock-out mutants. Although transient transfection studies have been reported in parasitic nematodes [16,17], they are still in their infancy and the established *C. elegans *system offers itself as a proxy model for functional studies of filarial promoters and antigens. Krause et al. [18] reported expression studies of *O. volvulus GST-1a *in *C. elegans *and Kampkötter et al. [19] have shown that *O. volvulus GST-3 *overexpressed in *C. elegans *could confer it increased resistance to oxidative stress. Similarly, a study by Redmond et al. [20] showed that transgenic *C. elegans *were able to express a glycosylated vaccine candidate protein of the gastrointestinal nematode of ruminants, *Haemonchus contortus*. Expression of vaccine candidate antigens in *C. elegans *could therefore represent a way forward to produce recombinant proteins that are correctly folded and bear nematode specific post-translational modifications, which might be relevant for inducing protective immune responses in vertebrate hosts.

*C. elegans *has been exploited as a heterologous transformation system to examine the activity and specificity of parasitic nematode gene promoters [21,22]. Beyond a mere role as an expression host, studies with transgenic *C. elegans *that express the reporter gene GFP under the control of the filarial cystatin promoter could also give an insight as to the localization and hence to the so far unknown roles of cystatin in nematodes. Apart from its role as an immunomodulator, cystatin has been hypothesized to regulate proteases that are involved in processes such as moulting, and it has been shown that inhibition of cysteine proteases with chemicals indeed interferes with moulting of *O. volvulus *[3,23]. It is therefore possible that the moulting process could be inhibited in *C. elegans *expressing filarial cystatin, or that another phenotype results. Moreover, expression and release of cystatin in transgenic parasitic nematodes could alter their interaction with the host, e.g. inducing an increased down-regulation of inflammatory immune responses.

As a prelude to such functional studies we analyzed the genomic organization of the cystatin of *A. viteae (Av17) *and defined its promoter elements. We further demonstrated that the *Av17 *promoter is functional in *C elegans *and compared the expression of the cDNA and the genomic sequence of *Av17 *in *C. elegans*.

## 2. Material and Methods

### 2.1 Maintenance of C. elegans strains

Wild type *C. elegans *(N2 var Bristol), phaI (e2123) mutants and transgenic worms were cultured on Nematode Growth Medium (NGM) plates seeded with *E. coli *OP50 and maintained as described [24]. Animals were cultured at 25°C (N2 var Bristol and transgenic worms) or at 15°C (phaI mutants).

### 2.2 Screening of an A. viteae genomic library and isolation of an Av17 clone

An *A. viteae *genomic library which was constructed in λ-dash was screened by DNA-DNA hybridization using a PCR product of 405 bp amplified from *Av17 *cDNA (GenBank Accession # L43053) with primers (Av17fw, 5'-GTT TTG GTG CGC TGT GAA-3'; Av17rv, 5'-CAC TGA TGA GAG TAC TTC-3') spanning the region from bp 89 to bp 493 of the cDNA. PCR amplification was performed under the following cycling conditions: 94°C for 5 min; 35 cycles of 94°C for 45 sec, annealing for 1 min at 58°C, and extension at 72°C for 2 min, with a final extension at 72°C for 10 min. The resulting product was purified by separation through a 1% agarose gel and DIG-labelled with the DIG High Prime DNA labelling Kit (Roche). Positive clones were re-screened by PCR using combinations of the λ-dash universal primers T3 and T7 and the Av17 reverse primer. Cycling conditions were: 94°C for 1 min; 30 cycles of 94°C for 45 sec, annealing for 1 min at 53°C, and extension at 72°C for 2 min, with a final extension at 72°C for 20 min. A product of approximately 2800 bp, which was amplified using T3 and the *Av17 *reverse primers was subcloned into pGEM-T Easy (Clontech) and sequenced.

### 2.3 Synthesis of A. viteae cDNA

To generate cDNA of *A. viteae*, worms were disrupted by grinding in liquid nitrogen and then suspended in lysis buffer (RNAeasy RNA Extraction Kit, Qiagen). Total RNA was extracted according to the manufacturers instructions. After RNA isolation, any residual DNA contamination was removed by digestion with RNAse-free DNAse (Promega). The RNA was precipitated to remove the DNAse, dissolved in RNAse-free water, and used as a template for oligo-(dT) primed reverse transcriptions, with Moloney Murine Leukemia Virus H (-) Point Mutant RT (Promega). The cDNA synthesized was used for PCR reactions with different primers.

### 2.4 Sequence analysis

Sequence analysis was performed using the ABI Dye Terminator chemistry. The cloned fragment of the *A. viteae *gDNA spanning the putative 5' upstream genomic sequence of cystatin was analyzed for the presence of promoter elements and transcription factor binding sites by using the *PromoterInspector *and *MatInspector *public software at  (Genomatix Software, Braunschweig, Germany).

### 2.5 Construction of Av17 promoter:reporter plasmids

For transfection of Cos7 cells, the pGEM-T plasmid containing the isolated genomic clone was used as a template in a PCR reaction to amplify the upstream genomic region using the plasmid-specific SP6 and a *Kpn*I linked clone-specific reverse primer (Av17rvKpn, 5' TAT CCG GTA CCA TCG TCG TTA GCT TTG TTT 3'). The product was digested with *Sac*I and *Kpn*I and ligated into pEGFP-N1 (Clontech). The resulting plasmid was digested with *Sac*I and *Afl*II. The 1710 bp fragment encoding the potential *Av17 *promoter, EGFP and the SV40-poly A was purified by agarose electrophoresis and ligated into pSL1180 (Pharmacia). To construct a promoter-less negative control plasmid, pEGFP-N1 was digested with *Eco*RI and *Afl*II. The 1010 bp fragment comprising the reporter gene and the SV40-polyA was then ligated into pSL1180. As positive control in transfection studies pEGFP-N1 was used.

To construct a *Av17 *promoter plasmid for the transformation of *C. elegans*, the pGEM-T plasmid containing the isolated genomic clone was used as a template in a PCR reaction to amplify the putative promoter region together with the first exon of *Av17 *using the specific primers (prAvfw, 5'-CCC AAG CTT TAA CCC TCA CTA AAG GGA-3' and prAvrv, 5'-AAC TGC AGA TTG CGT TCC TGC CAT CC-3'). The product was cloned into the vector pPD 95.77 (Fire lab kit, 1995), in-frame with the reporter gene GFP to obtain pPrAvGFP.

### 2.6 Construction of plasmids for expression of Av17 in C. elegans

The *Av17 *cDNA and the pGEM-T clone containing the genomic sequence of *Av17 *were used as templates in PCR reactions with primers that incorporated a *Nhe*I restriction site at the 5' end of the sequence (*Av17*fwNhe, 5'-TAT TCA GGT AGC ATG ATG TTG TCA ATA AAG-3') in conjunction with a primer that incorporated a C-terminal 6X His tag and a *Kpn*I restriction site at the 3' end (*Av17*rvHisKpn, 5'-TAT TCA CGG TAC CTC AAT GGT GAT GGT GAT GGT GAT GCA CTG ATG AGA GTA C-3'). The PCR products were cloned into the vector pPD49.83, which contains the *C. elegans hsp16/41 *promoter and a synthetic intron, to obtain p49Av17c (*Av17 *cDNA) and p49Av17g (*Av17 *gDNA). Protein expressed under the control of this promoter on heat shock is targeted to the gut cells of transgenic worms [25].

The cDNA sequence of *Av17 *was also amplified using the specific primers, 5'-TAT TCA GCT AGC ATG CAC CAT CAC CAT CAC CAT ATG ATG TTG TCA ATA AAG-3' (Av17fwHisNhe) incorporating NheI sequence and an N-terminal 6XHIS tag and 5'-TCC CCC CGG GTC ACA ATG TAC TTT A-3' (Av17rvUTRSma) incorporating the 3'UTR of the Av17 gene and *Sma*I restriction site. The product was cloned into the vector pPD103.05 to obtain p103Av173'. This plasmid was chosen since the *let-858 *promoter ubiquitously expresses in all *C. elegans *tissues [26].

### 2.7 Transformation of Cos7 cells

Cos7 cells were maintained in RPMI 1640 (Biochrom) supplemented with 10% FCS (Biochrom) under standard tissue culture conditions and were transfected using lipofectamin reagent (Invitrogen). Cells were plated out (10^5 ^per well) in 12-well plates 24 h prior to transfection. Two μg of the desired plasmid and 8 μl lipofectamin were resuspended in 100 μl serum-free medium separately. After 30 min incubation at room temperature (RT) the solutions were mixed and incubated for another 30 min at RT. To this, 800 μl serum-free medium was added and applied to the cells. After 6 h of incubation under standard tissue culture conditions the transfection reagent was removed and the cells were maintained in complete RPMI 1640 before microscopic examination after 48 h.

### 2.8 Transformation of C. elegans

For promoter studies, young adult hermaphrodite wild-type (WT) worms were immobilized on agarose pads overlayed with mineral oil and the construct pPRAvGFP along with the marker plasmid pRF4 (a kind gift from James M. Kramer, Northwestern University Medical School) was microinjected into the gonads at a concentration of 100 ng/μl. The injection was done at a pressure of 460 psi using femtotips (Eppendorf, Germany). The transformation buffer contained 2% w/v PEG 6000, 20 mM KH_2_PO_4 _and 3 mM potassium citrate. The marker plasmid confers transgenic worms with a roller phenotype. Only worms of the second and subsequent generations, which showed both the roller phenotype and GFP expression were used for further analysis of the *Av17 *promoter. As a negative control, WT worms were microinjected with pRF4 and promoter-less pPD95.77.

For the expression of Av17, *C. elegan*s, pha-1mutants (e-2123) were transformed by microinjecting the constructs p49Av17c, p103Av173' and p49Av17g into the gonads together with the marker plasmid pBX at a concentration of 100 ng/μl each. The transformed worms were selected by their ability to survive and reproduce at 25°C. The selected worms 103cAv173', 49cAv17 and 49gAv17 were maintained as discrete lines at 25°C. Integration of the extrachromosomal arrays was achieved by gamma irradiation of the transgenic worms with 35 Gy. The progeny of these worms was then screened for 100% transmittance to obtain lines with chromosomally integrated transgens. To verify if the transformed *C. elegans *contained the Av17 construct, single worm PCR was done on the F2 generation using the same primers as for the RT-PCR. Single worms were picked from NGM plates and suspended in 2.5 μl of lysis buffer (50 mM KCl, 10 mM Tris-HCl pH 8, 2.5 mM MgCl_2_, 0.45% w/v Nonidet P 40. 0.45% v/v Tween 20, 0.1% gelatin and 200 μg/ml Proteinase K) and frozen on dry ice for 20 min. The samples were then incubated at 60°C for 1 h for protein digestion and at 95°C for 15 min to inactivate Proteinase K. The resulting DNA was mixed with PCR master mix, cycling conditions were 94°C for 5 min, followed by 35 cycles of 94°C for 1 min, 57°C for 1 min, and 72°C for 1 min and a final extension of 72°C for 10 min. The products were resolved on 1% agarose gel.

### 2.9 Microscopy

Light and fluorescence microscopy observations for EGFP detection in Cos7 cells were made using a Zeiss Axioplan fluorescent microscope (Oberkochen, Germany) with a blue (number 487909) excitation filter set. The transgenic *C. elegans *worms with promoter-reporter constructs were immobilized on agarose pads and observed using a Leica Confocal Microscope (TCS SP2) (Heidelberg, Germany).

### 2.10 Analysis of DNA transcription

Transcription of the injected construct was analyzed by RT-PCR using total RNA prepared from 0.5 mg wet weight of transformed worms (103Av173', 49cAv17 and 49gAv17) as described above. Transgenic lines 49cAv17 and 49gAv17 were given a heat shock at 33°C for 3 h before total RNA isolation. The synthesized cDNA was used as template in a PCR reaction using Av17 specific primers (Av17fwNhe and Av17rv, see above). Amplification from non-transgenic worms was included as control. PCR was performed with the following thermal cycling conditions: 94°C, 1 min, 30 cycles of 94°C for 1 min, annealing 1 min 57°C, and extension at 72°C for 2 min, final extension 72°C for 10 min. The products were separated on 1% agarose gel.

### 2.11 Analysis of the expression of Av17 in transgenic C. elegans lysate

Transgenic worms were grown in large numbers in NGM liquid cultures at 25°C with *E. coli *OP50 as a source of food. Worm line with the Av17 gene downstream of the inducible *hsp *promoter (49cAv17) was given a heat shock at 33°C for 3 h. The main contaminants from the nematode culture were cleaned by centrifugation through 60% sucrose in 0.1M NaCl [27]. The worms were frozen in liquid nitrogen and homogenized with a mortar and pestle in lysis buffer (20 mM NaH_2_PO_4_, 300 mM NaCl, pH 7.4). The lysate was separated on a 14% SDS-polyacrylamide gel. Western Blot analysis was performed as described [28]. The blot was incubated with a 1:5000 dilution of monoclonal anti-His mouse antibody (Qiagen, Germany) and alkaline phosphatase coupled goat anti-mouse antibody diluted 1:5000 was used as conjugate.

Furthermore, the progeny of the transgenic worms expressing Av17 constitutively (103cAv173') was quantified and compared to the uninduced transgenic worms with Av17 downstream of *hsp *promoter (49cAv17). Young adult hermaphrodite (six of each) worms were transferred to NGM plates and the number of eggs and of larvae at every molt were quantified.

## 3. Results

### 3.1 Isolation of a genomic Av17 clone

**Figure 1:**
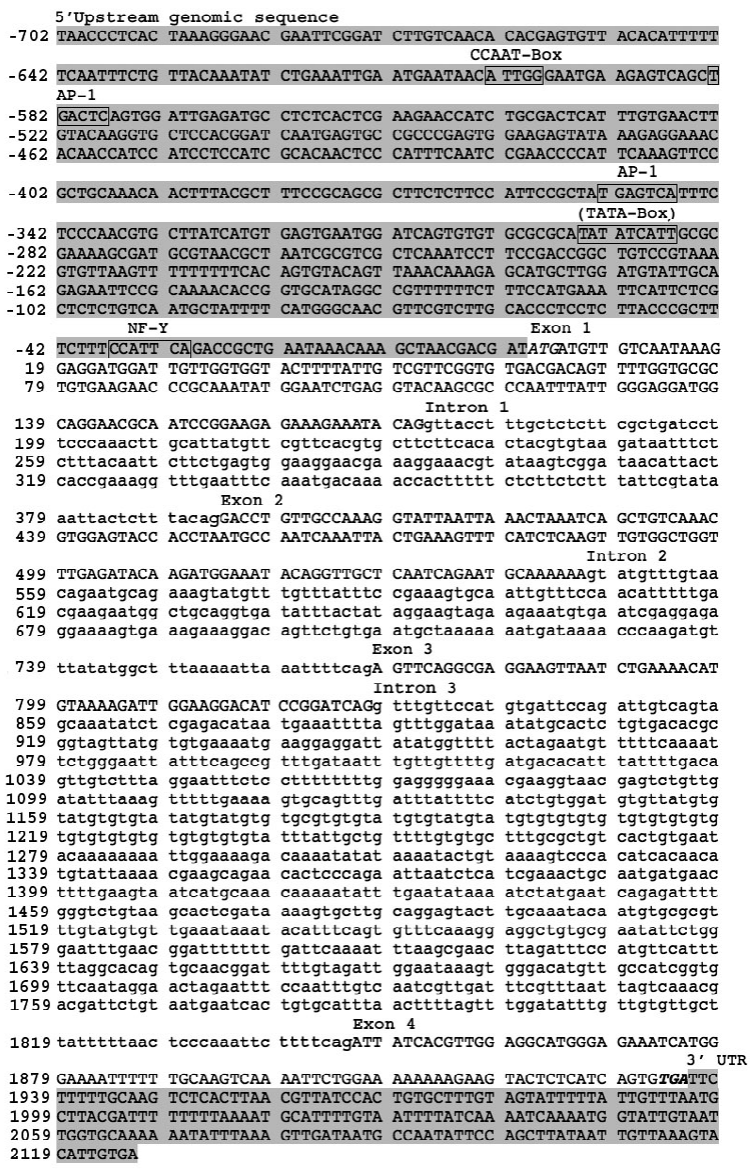
The nucleotide sequence of *Acanthocheilonema viteae *cystatin gene showing the 5' upstream genomic sequence, four exons interrupted by three introns and the 3' UTR. The putative TATA box and transcription factor binding sites (CCAAT box, two AP-1 binding sites (CCAAT) and one NF-Y binding site (TGAGTCA)) are boxed. The sequence highlighted in gray shading corresponds to the 5' upstream genomic sequence and 3' UTR. Exons are in capital letters. The start ATG and the stop TGA are italizised.

After screening of ~10,000 plaques of an *A. viteae *λ-dash genomic library, a positive clone (gAv17) was obtained. The insert of gAv17 was ~2.8 kb in length, and when sequenced it was found to include the entire *Av17 *gene. Comparison to the published cDNA sequence of the *A. viteae Av17 *revealed that the genomic sequence is comprised of 702 bp of the putative promoter region, 4 exons of 166 bp, 158 bp, 51 bp and 97 bp, interspersed by 3 introns of sizes 222 bp, 158 bp and 1020 bp, followed by 191 bp of 3' untranslated sequence (Fig. 1).

Several putative regulatory sequences could be identified by computer analysis in the 702 bp upstream genomic sequence (Fig. 1). A putative TATA-box was located at position -296. Consensus recognition sites for the transcription factors NF-Y and AP-1 were identified at positions -37, -353 and -583, respectively. An inverted CCAAT box was located at position -603.

### 3.2 Functional studies of Av17 promoter

The putative promoter region of 702 bp was capable of promoting transient expression of the reporter EGFP in mammalian cells. EGFP fluorescence was detected in 5 % of Cos7 cells transfected with the promoter-EGFP construct (data not shown) with the same intensity as the positive control. Cos7 cells transfected with a promoter-less control plasmid did not show any EGFP fluorescence.

### 3.3 Promoter studies in transgenic C. elegans

**Figure 2:**
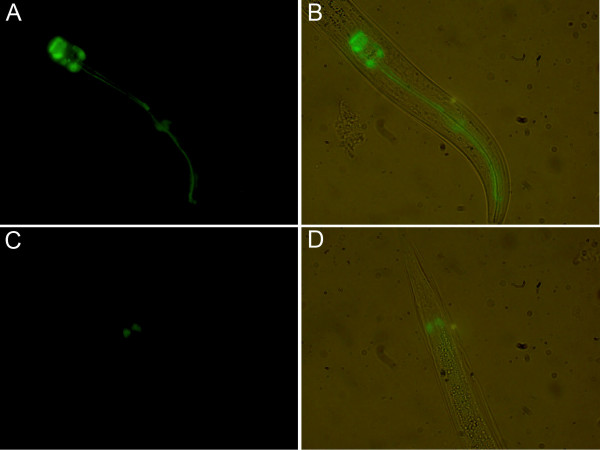
Expression pattern of the *Av17promoter*::GFP construct in transgenic *Caenorhabditis elegans*. (A) Fluorescence staining in the pharynx of a worm transformed with the Av17promoter::GFP fusion construct. The pharynx contains two classes of gland cells, g1 (two cells) and g2 (two cells) in the second bulb of the pharynx. The g1 cells extend three cuticle-lined ducts anteriorly within the narrow pharyngeal nerve cords, two of which pass through the isthmus and empty into the pharyngeal lumen near the first bulb and the dorsal g1 duct empties near the anterior end of the pharynx. The g2 cells extend shorter ducts, which empty into the lumen of the second bulb. (C) Fluorescence staining in the rectal gland cells (rectD, rectVL, rectVR) in the posterior of the same worm as in (A). These cells connect to the intestinal lumen just posterior to the rectal valve. (B) and (D) Bright field microscopy of (A) and (C), respectively.

*C. elegans *was used as an heterologous system to analyze the ability of the upstream genomic sequence of Av17 to promote the expression of the reporter gene, GFP. The promoter construct was microinjected into the gonads of young wild type *C. elegans *together with the marker plasmid. Transgenic animals from three transformed lines were examined. All the lines showed GFP expression in all stages of the worms, L1, L2, L3 and adults, but not in the eggs. Strongest expression was observed in the gland cells of the pharynx, which consist of 2 cells (g1 and g2; Fig. 2A,B). Expression of GFP was also observed in the rectal glands (Fig. 2C,D), which consists of three large cells (recD, recVL, rectVR) and are connected to the intestinal lumen just posterior to the rectal valve.

### 3.4 Transformation of C. elegans with A. viteae cystatin

**Figure 3:**
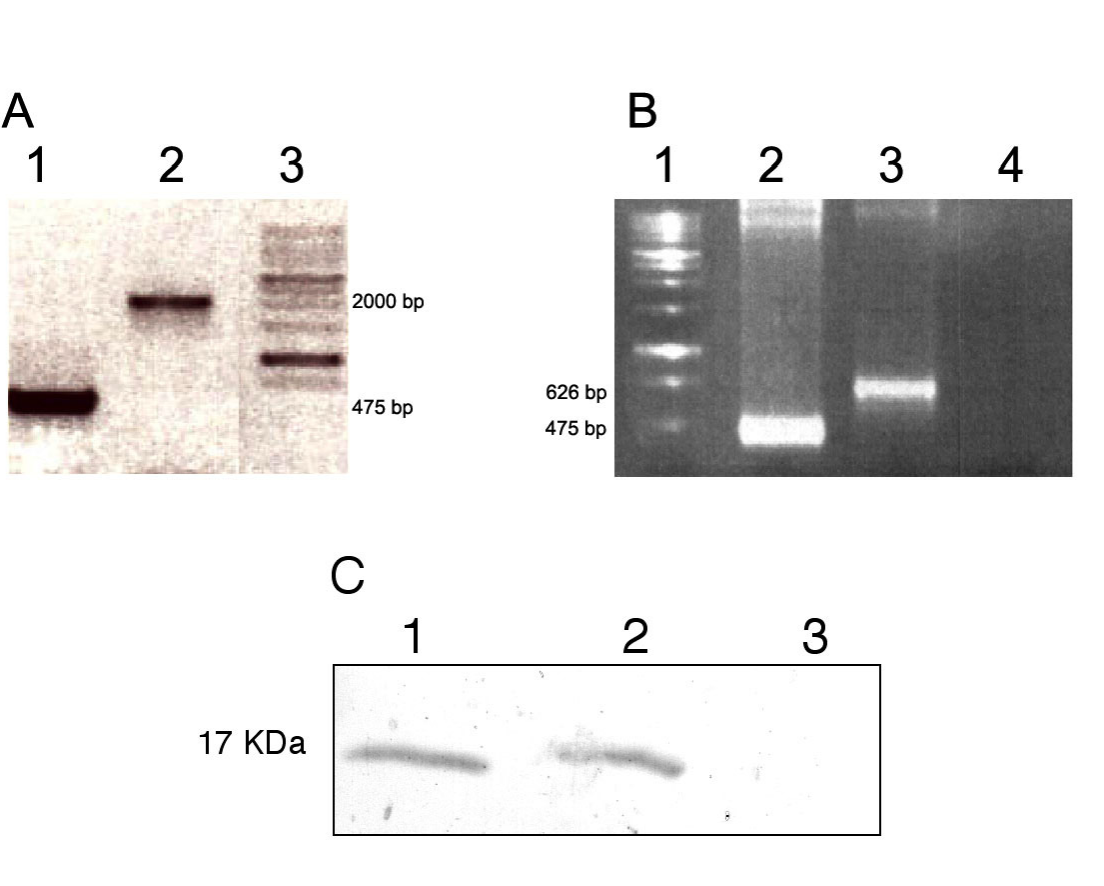
(A) Single worm PCR of transgenic worms, 49cAv17 and 49gAv17, containing the cDNA (lane 1) and genomic sequence (lane 2) of *A. viteae *cystatin respectively. (B) RT-PCR analysis of transcripts from transgenic worms, 49cAv17 and 49gAv17, containing the cDNA (lane 2) and genomic sequence (lane 3) of *A. viteae *cystatin respectively. Negative control with wild type *C. elegans *in lane 4 and marker in lane 1. (C) Western Blot with anti-His antibodies. Lane 1: Transgenic *C. elegans *expressing Av17 constitutively (103cAv173') ; Lane 2: Transgenic *C. elegans *(49cAv17) expressing Av17 induced by heat shock ; Lane 3: pha-1 control worms

The temperature sensitive pha I mutant of *C. elegans *was used for microinjection experiments to facilitate selection of the transgenic worms expressing Av17. The transcription factor pha-1 is required for the morphogenesis of the pharynx [29,30]. In the pha-1 mutant worms the pharynx fails to undergo differentiation and the mutation interferes with the embryonic development at 25°C making it easier for the selection of transgenic worms, that are co-transfected with the rescue plasmid. Transgenic worms lines, 103cAv173' (injected with p103Av17), 49cAv17 (injected with p49Av17c) and 49gAv17 (injected with p49Av17g) were established and maintained as discrete lines. In order to integrate the plasmid constructs into the genome, worms were exposed to γ-irradiation and subjected to another round of screening. The F2 and subsequent generations of transgenic lines were used for further analysis. Single worm PCRs to test for the presence of injected constructs showed that the worms (49cAv17, 103cAv173' and 49gAv17) were transgenic. No product was amplified from the pha-1 control worms (Fig. 3A).

### 3.5 Transcription and translation of Av17 in C. elegans

**Figure 4:**
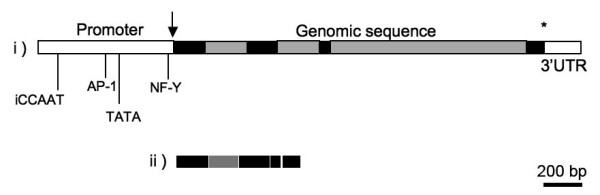
i) Schematic representation of the genomic structure of Av17 (black and grey boxes represent the exons and the introns respectively). ii) intron splicing of genomic sequence of Av17 *C. elegans *(103cAv173').

The transcription of *Av17 *in *C. elegans *was shown by RT-PCR. A 475 bp product consistent with the predicted size of the coding region was obtained from the clones containing the cDNA sequence of *Av17 *(Fig. 3B, lane 2). In worms with the genomic sequence (49gAv17), transcripts of a larger size were amplified, which on sequencing were found to include a part of the first intron (Fig. 3B, lane 3). Further analysis revealed that the conserved intron splice donor site AG/gt was apparently read through and the second AG/gt downstream in the first intron was recognised as the splice donor site. The remaining introns were spliced out correctly to obtain a transcript of 626 bp (Fig. 4, ii) including 152 bp of the first intron, instead of a transcript size of 475 bp, if splicing had occurred correctly. No amplification product was obtained from the control pha-1 worms.

In a western blot, anti-His antibodies specifically recognized a band of 17 kDa in the transgenic *C. elegans *lines 103cAv173' and 49cAv17 expressing Av17 constitutively and under heat shock (Fig. 3C). No expression was seen in pha-1 control worms and 49gAv17.

In worms expressing Av17 constitutively (103cAv173') of 20 ± 15.9 eggs only 5.2 ± 3.8 developed to adults. The reduction in the ability of the eggs to develop to adults is significant (p < = 0.0129) when compared to worms transformed with Av17 downstream of hsp promoter but uninduced, where of 24 ± 16.4 eggs, 19 ± 10.2 developed to adults (data not shown).

## 4. Discussion

In this study, we have determined the entire genomic organization and the sequence of *A. viteae *cystatin gene. We identified putative promoter elements in the upstream genomic sequence and used *C. elegans *as a heterologous system to determine the localization of Av17 in the worm tissues. We have also expressed the parasite protein in *C. elegans*, demonstrating that it is possible to use free-living nematode as a system for the expression of parasitic nematode proteins.

To date, we have isolated and characterized one cystatin gene from *A. viteae*, although 2 isoforms of cystatin have been reported in *B. malayi *[6] and in *C. elegans *[13]. In *C. elegans *the cystatins are also secreted as in the parasitic worms but they do not have the same immunomodulatory properties [13], instead they could play a role during moulting. RNAi of *C. elegans *cystatins does not show visible phenotypes, possibly because their loss of function is compensated by other protease inhibitors.

The genomic sequence of *Av17 *contains 3 introns of sizes 222 bp, 158 bp and 1020 bp respectively. All of the introns in the *Av17 *gene have a consensus splice donor site, gt and a consensus splice acceptor site, AG [31]. The 702 bp upstream genomic sequence of *Av17 *contains a TATA box, an inverted CCAAT box sequence and consensus recognition sites for the transcription factors AP-1 and NF-Y. Binding sites for the transcription factor AP-1 are found in numerous mammalian immunoregulatory and inflammatory genes [32]. In the promoter region of the mammalian salivary Cystatin S the presence of an AP-1 binding site has been suggested to play a role in the regulation of expression [33]. Among parasites, AP-1 has been shown to be involved in the transcriptional regulation of *Schistosoma mansoni *calreticulin [34]. Upstream genomic sequences of *C. elegans *cystatins do not have transcription factor binding sites comparable to *Av17*. Additional studies on the promoter elements are required to determine the role they play in the regulation of cystatin. The putative promoter sequence was however able to drive GFP expression in Cos7 cells, demonstrating that it is functional.

The expression pattern of Av17 was analyzed in transgenic lines of *C. elegans *obtained by microinjection of the Av17promoter::GFP construct. We observed GFP expression in all larval stages and the adults of the transgenic *C. elegans*, showing that Av17 is temporally expressed as in the parasitic worms [4]. The strongest expression was observed by light microscopy in the g1 and g2 gland cells of the pharynx and the rectal gland cells of transgenic *C. elegans*. However, immunostaining by indirect immunofluorescence using anti-Av17 antibodies localized Av17 in *A. viteae *and using anti-*C. elegans *cystatin antibodies localized cystatins in *C. elegans *to the hypodermis and in developing oocytes and embryo (unpublished own data). It is possible that the expression in pharyngeal and rectal glands also occurs in filarial worms, but as these nematodes cannot be transformed with reporter gene constructs as yet, an analysis of expression in these glands would require extensive ultrastructural studies. The immunolocalization by indirect immunofluorescence in the hypodermis and in developing stages, i. e. in compartments that were not stained by reporter gene expression, suggests immunolocalization might be more sensitive than reporter gene expression. Alternatively, it is possible that the *Av17 *promoter sequence used may be incomplete and requires additional regulatory elements to drive stronger expression to the hypodermis. The inability of the *C. elegans *transcription machinery to read filarial regulatory sequences correctly also cannot be ruled out. Future expression analysis of the promoter sequence of *C. elegans *cystatins would resolve this.

The expression of the reporter gene in pharyngeal and gland cells, i.e cells that release products to the exterior environment, is compatible with the earlier shown and suggested functions of cystatin as an immunomodulator [13] and in moulting [37], respectively. As these gland cells are stimulated synchronously with pharyngeal pumping activity, the question arises whether cystatin has a role in digestion. Generally, in *C. elegans *the gland cells have been proposed to store digestive enzymes since digestion and absorption of nutrients take place in the gut. However, in filarial nematodes transcuticular transport is regarded as the main route for the absorption of nutrients [36]. This suggests that Av17 in filarial nematodes may not be involved in the regulation of digestive proteases. Instead filarial cystatin may have evolved to become an immunomodulator that is permanently secreted by the parasites. This would be in line with the observation that molecules of the filarial worms have the capacity to induce the production of anti-inflammatory cytokines in host macrophages and change the phenotype of these cells, while *C*. *elegans *cystatins do not have these properties [13].

Since cystatin is a potential vaccine candidate [14], we aimed to use *C. elegans as *expression system. A possible advantage of using *C. elegans *as a host for expression of filarial candidate vaccine antigens, compared to procaryotic expression systems, is that post-translational modifications, which might be relevant for inducing protective immune responses, would likely be conserved among the nematodes. *A. viteae *cystatin is predicted to have one O-glycosylation and two phosphorylation sites. The DNA and the amino acid sequence of Av17 differs considerably from the *C. elegans *cystatins except at the evolutionarily conserved enzyme binding site [13]. Additionaly *C. elegans *is a suitable system for evaluating its potential to express this candidate vaccine antigen since detection of the recombinant protein is possible by specific antibodies.

The expression of Av17 was detectable in transgenic *C. elegans *lines transformed with the cDNA sequence by immunoblotting with anti-His antibodies and antibodies specific for Av17 although, we were unable to purify the protein using Ni-chelate affinity chromatography. This is probably due to inaccessibility of the 6 His-leader in lysates, an effect that is not uncommon for other recombinant proteins (own unpublished observations) and depends probably on the interaction of the charged 6-His tag with other stretches of the protein. An increase of the expression of recombinant Av17 and the use of other purification methods could lead to production of *C. elegans*-expressed Av17 in amounts amenable to further studies. Furthermore, the reduction in the number of eggs developing to adults in worms expressing Av17 constitutively suggests that the expression of protease inhibitor interfered with proteases involved in the differentiation or moulting of the worms.

In the *C. elegans *lines, 49gAv17, *Av17 *transcripts included a part of the first intron. A sequence 152 bp downstream in the first intron was recognised as the 5' splice donor site and has the consensus sequence of AG/gt. It has a guanine (g) at the +5 position (i.e., AG/gtttga), unlike the original donor splice site of the first intron of Av17, which has a cytosine (i.e., AG/gttacc). Introns 2, 3 and 4 have a guanine at the +5 position (Table 1). Most *C. elegans *introns [38] also have this conserved guanine at the +5 position of the donor site including those of the homologous cystatins. This suggests that the conserved G at the +5 position in the donor splice site might be necessary to be recognised as intron 5' site in *C. elegans*. Most of the introns (70%) of another gene of *A. viteae*, chitinase, have the conserved G at the +5 position (Babila Tachu, personal communication), but there is not enough information on the organisation of the genes of this filarial species to allow a conclusion on the specificity of exon/intron recognition. The 3' splice intron acceptor sequence of uucag/A was recognised correctly and the other introns were spliced at the normal sites as in *A. viteae*. Earlier studies with the expression of *O. volvulus *and *H. contortus *proteins as GFP fusions in *C. elegans *showed that the introns were spliced out normally [18,20]. Our results for the first time suggest that the intron splicing and recognition may differ among *C. elegans *and *A. viteae *at least with respect to cystatin. Owing to such differences, the expression of foreign genes in *C. elegans *might be more successful if cDNA sequences of genes are used, as compared to the use of genomic DNA.

**Table 1:** Intron splice donor and acceptor sites of Av17 in *A. viteae *and in transgenic *C. elegans*.

Intron	Size	5' Splice donor site of cystatin in *A. viteae*	5' Spilce donor site of *A. viteae *cystatin recog-nised in *C. elegans*	3' Splice acceptor site
1	221 bp	ATACAG/gttacct	CGAAAG/gtttga	ttacag/GACCTG
2	220 bp	AAAAAA/gtatgtt	AAAAAA/gtatgtt	tttcag/AGTTCA
3	1017 bp	GATCAG/gtttgtt	GATCAG/gtttgtt	tttcag/ATTATC

## Authors' contributions

SP carried out the cloning, promoter studies and expression in *C. elegans *and drafted the manuscript. BK worked on the genomic sequence, analysed the promoter and contributed to the manuscript. EL was involved in the generation of transgenic *C. elegans*. SH contributed to the revising of the manuscript. FT was involved in the generation of transgenic *C. elegans*. RL conceived the study, and participated in its design and coordination and helped to draft the manuscript. All authors read and approved the final manuscript.
